# Timing and Duration of Prediabetic Metabolic Dysfunction Differentially Impact Cerebral Perfusion, Vascular Structure, and Behavior

**DOI:** 10.1007/s12265-026-10806-3

**Published:** 2026-08-05

**Authors:** Annika Matthiesen, Stephanie DiLucia, Riley Feagle, Sadie Lofton, Isabel Marshall, Catrina Robinson, Adviye Ergul

**Affiliations:** 1 Medical University of South Carolina, Charleston, SC USA; 2 Midwestern University, Glendale, AZ USA; 3 Ralph H. Johnson VA Health Care System, Charleston, SC USA

**Keywords:** Obesity, Cerebrovascular function, Dementia, Vascular changes

## Abstract

Obesity is a major risk factor for metabolic dysfunction and cognitive decline, yet the influence of age of onset and sex on neurovascular outcomes remains unclear. We examined how the age at initiation and duration of diet-induced obesity influence metabolism, cognition, and cerebrovascular perfusion in male and female mice. A high-fat diet increased body weight and impaired glucose regulation, particularly in females. Obesity selectively impaired recognition memory while sparing spatial working memory. Cerebrovascular effects were region- and age-specific. Early- and mid-life obesity produced localized perfusion changes despite preserved global cortical blood flow. In contrast, early-life obesity was associated with reduced cortical perfusion and decreased hippocampal vascular complexity, including fewer branch points and smaller vessel diameters. These alterations parallel recognition memory deficits, suggesting a vascular mechanism linking early metabolic stress to cognitive impairment. Together, our findings identify early-life obesity as a critical window of vulnerability for long-term neurovascular and cognitive dysfunction.

**Supplementary Information:**

The online version contains supplementary material available at 10.1007/s12265-026-10806-3.

## Introduction

Obesity is a growing global health concern and a well-established risk factor for metabolic disorders, including type 2 diabetes and cardiovascular disease. Increasing evidence also implicates obesity as a modifiable risk factor for cognitive decline and dementia later in life [1, 2]. Epidemiological studies link early- and mid-life obesity to increased risk of Alzheimer’s disease and related dementias [3, 4], yet the biological mechanisms underlying this association remain poorly defined. Age of onset [3, 4], diet quality [5], and sex [6] independently influence dementia risk, but how these factors interact to shape brain and cerebrovascular health remains unclear.

Obesity-related cognitive impairment is often attributed to metabolic dysregulation, including insulin resistance and impaired glucose homeostasis, which can adversely affect neuronal function and brain energy metabolism [7, 8]. However, obesity is also associated with cerebrovascular dysfunction, including altered cerebral blood flow, endothelial dysfunction, and disruption of the blood–brain barrier [9–11]. Because vascular alterations are among the earliest pathological changes observed across dementia subtypes [12], cerebrovascular vulnerability may represent a critical link between systemic metabolic dysfunction and cognitive decline. Yet, how diet-induced obesity affects cerebral perfusion and vascular structure across the lifespan remains unclear.

Sex is an important but underexplored biological variable in metabolic and neurodegenerative research. Males and females differ in adipose tissue distribution, metabolic regulation, and susceptibility to insulin resistance [13–15], but many preclinical studies examining obesity-related cognitive decline have focused primarily on males [16]. Understanding sex-specific vulnerability across the lifespan is therefore essential.

Age of obesity onset may further shape long-term neurological outcomes. Early-life obesity has been associated with lasting cognitive impairments and increased dementia risk [17], with obese individuals in young adulthood exhibiting a nearly threefold increased risk compared to age-matched controls [18]. Whether early exposure produces more severe and persistent cerebrovascular deficits, however, remains unclear.

To address these gaps, we systematically examined diet-induced obesity across early-life, young adulthood, and mid-life, with an emphasis on sex differences and temporal dynamics. We combined metabolic, behavioral, and blood flow assessments to determine how the timing and duration of obesity influence brain function and cerebrovascular integrity, and to identify potential windows of vulnerability relevant to dementia risk.

## Methods

###  Animals

Male and female C57Bl/6J mice were purchased from the Jackson Laboratory (Bar Harbor, Maine). Mice began either a standard diet (STD; 10% kcal from fat, Research Diets Inc.; #D12450B, New Brunswick, NJ) or a high-fat diet (HFD; 54% kcal from fat, #D05090701) at 4 or 16 weeks of age. Diets were consumed for 24 weeks, and weight was measured every 4th week. The animal protocol was approved by the Ralph H. Johnson VA Medical Center Research and Development Committee (NURD-010–21 F, ACORP 712).

###  Fasting Glucose Measurement

Mice were fasted for 4 h with free access to water. Baseline blood glucose levels were measured via tail vein puncture using a handheld glucometer (AlphaTrak3), followed by administration of a 1 g/kg glucose load. Subsequent blood glucose levels were measured 15 min, 30 min, 60 min, and 2 h post-injection. All measurements were performed under consistent conditions to minimize circadian and stress-related variability.

### Behavioral Assessments

Behavior was tracked using Noldus EthoVision XT 17. Novel Texture Recognition (NTR) Task was conducted as previously described [19]. Briefly, mice were exposed to two identical sandpaper stimuli (80- or 100-grit) for 5 min, with grit assignment randomized across groups. After a 1-hour delay, one familiar and one novel texture were presented. Recognition was calculated as the percentage of exploration time directed towards the novel texture.

For Novel Object Location (NOL) task, mice explored two objects during familiarization; in testing, one object was relocated. NOL performance was quantified as the percentage of exploration time directed toward the novel location. Total memory performance was calculated as the percentage of exploration time spent at novel and previous object locations relative to total exploration time.

The Y Maze task was conducted over three days (habituation, training with one arm closed, and testing with all arms open). Spatial memory was assessed by novel arm entries, time spent in the novel arm, and time spent in the center.

Across tasks, mice showing > 70% baseline preference were excluded.

Z-scores were calculated to further control for variation. z=(X − µ)/σ, where *X* represents the individual value, *µ* is the control group mean, and *σ* is the standard deviation of the control group.

###  Cortical Perfusion

Cortical blood flow was assessed using Laser Speckle Contrast Imaging. Mice were anesthetized with ketamine/xylazine (60–80 mg/kg and 7.5–10 mg/kg), and the intact skull was exposed via midline incision. Imaging was performed via Perimed PeriCam PSI positioned at a fixed distance above the skull. Cerebral perfusion was continuously recorded for 1 min (one image every 10 s), and values were averaged across 6 images per animal.

Global perfusion was calculated across the visualized cortex. Regional perfusion was assessed using six predefined regions of interest (frontal, parietal, temporal, and occipital cortices) aligned to the Allen Brain Atlas (Fig. 4d), which were customized to each animal to calculate mean perfusion per unit area (mm²). To account for inter-cohort variability, perfusion value was normalized to the corresponding control group mean.

###  Immunohistochemistry

Mice underwent transcardial perfusion with 2% paraformaldehyde, and brains were post-fixed and sectioned into 100 μm coronal slices using a vibratome. Vascular labeling was performed using DyLight 594-conjugated lectin (Abcam, L32471) at a 1:200 dilution. Sections were incubated with the lectin solution overnight at 4 °C under gentle agitation. Slices were mounted using a DAPI-containing mounting medium (ThermoFisher P36931) to visualize nuclei.

###  Vascular Imaging and 3D Reconstruction

Leptin-stained hippocampal/dentate gyrus vasculature was imaged using a Zeiss LSM 880 confocal or Keyence BZ-X710 to generate 3D z-stacks at 20×–25× magnification (water immersion lens). Images were analyzed with Imaris 10.1 to quantify branch points, mean segment diameter, and vascular volume fraction (vascular surface volume/total image volume ×100). Four coronal sections per animal were analyzed, and values were averaged.

###  Statistical Analysis

Statistical analyses were performed using Prism, version 9.3.1 (GraphPad Software, Inc). Data were analyzed via two- or three-way analysis of variance (ANOVA) with post-hoc comparisons or unpaired t-test as described in corresponding figure legends. Data represented as mean ± standard deviation (SD), plotted as biological replicates (n). Significance established at an α level of 0.05.

## Results

### Diet-Induced Obesity Produces Sex-Dependent Alterations in Glucose Metabolism

**Fig. 1:**
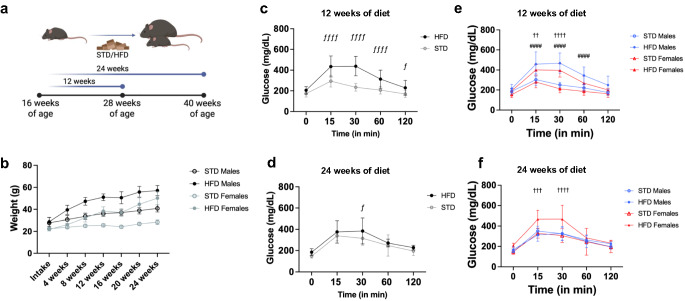
Diet-Induced Obesity Produces Sex-dependent Alterations in Glucose Metabolism. Schematic of experimental paradigm (**a**) and weight gain (**b**). Glucose tolerance test (GTT) after 12 (**c**) and 24 weeks of diet (**d**). Sex specific GTTs after 12 (**e**) and 24 weeks of diet (**f**). Data represented as mean ± SD. Data were analyzed using two-way ANOVA with time on diet and diet as factors, followed by Šídák’s multiple comparisons test (**b**-**d**) or three-way ANOVA with diet, sex, and time as factor following Tukey’s Honestly Significant Difference (HSD) Test (**e**-**f**). *p* < 0.05 (*), *p* < 0.01 (**), *p* < 0.001 (***), *p* < 0.0001 (****). ƒ, †, and # indicate post hoc comparisons for combined-sex, female-only, and male-only groups, respectively; the number of symbols reflects the same *p*-value thresholds as asterisks. n $\:\ge\:$ 7

At 16 weeks of age, animals were placed on either a HFD or STD and maintained for 12 or 24 weeks (Fig. 1a). Across both time points, HFD significantly increased body weight in both male and female mice relative to sex- and age-matched controls, with significant effects of diet (F(6,278), *P* < 0.0001) and sex (F(1,278) = 748.4, *P* < 0.0001; Fig. 1b). After 12 weeks of diet, HFD animals exhibited significantly elevated circulating glucose levels compared to controls (F(4, 160) = 9.488 (interaction), 51.66 (time), 122.3 (diet); Fig. 1c) following a glucose tolerance test (GTT). Post hoc analysis revealed increased glucose levels at all post-baseline time points. In contrast, after 24 weeks of diet, glucose intolerance was attenuated, with significant differences observed only at the 30-minute time point despite main effects of time and diet (F(4, 165) = 52.16 (time), 14.44 (diet); Fig. 1 d).

Time, sex, and diet significantly contributed to glucose variability at both 12 (Fig. 1e) and 24 weeks (Fig. 1f), with a significant sex × diet interaction detected only after 24 weeks of diet (p $\:\le\:$ 0.0001; F(1, 155) = 19.12; Fig. 1f). Post hoc analyses revealed that both males and females exhibited elevated glucose levels at 15 and 30 min after 12 weeks of diet (Fig. 1e); however, sustained impairment was observed exclusively in females after 24 weeks, which displayed reduced glucose clearance at the 15- and 30-minute time points following glucose injection (Fig. 1f).

### Task-Specific Behavioral Deficits in NOL and NTR are Driven by Diet

**Fig. 2:**
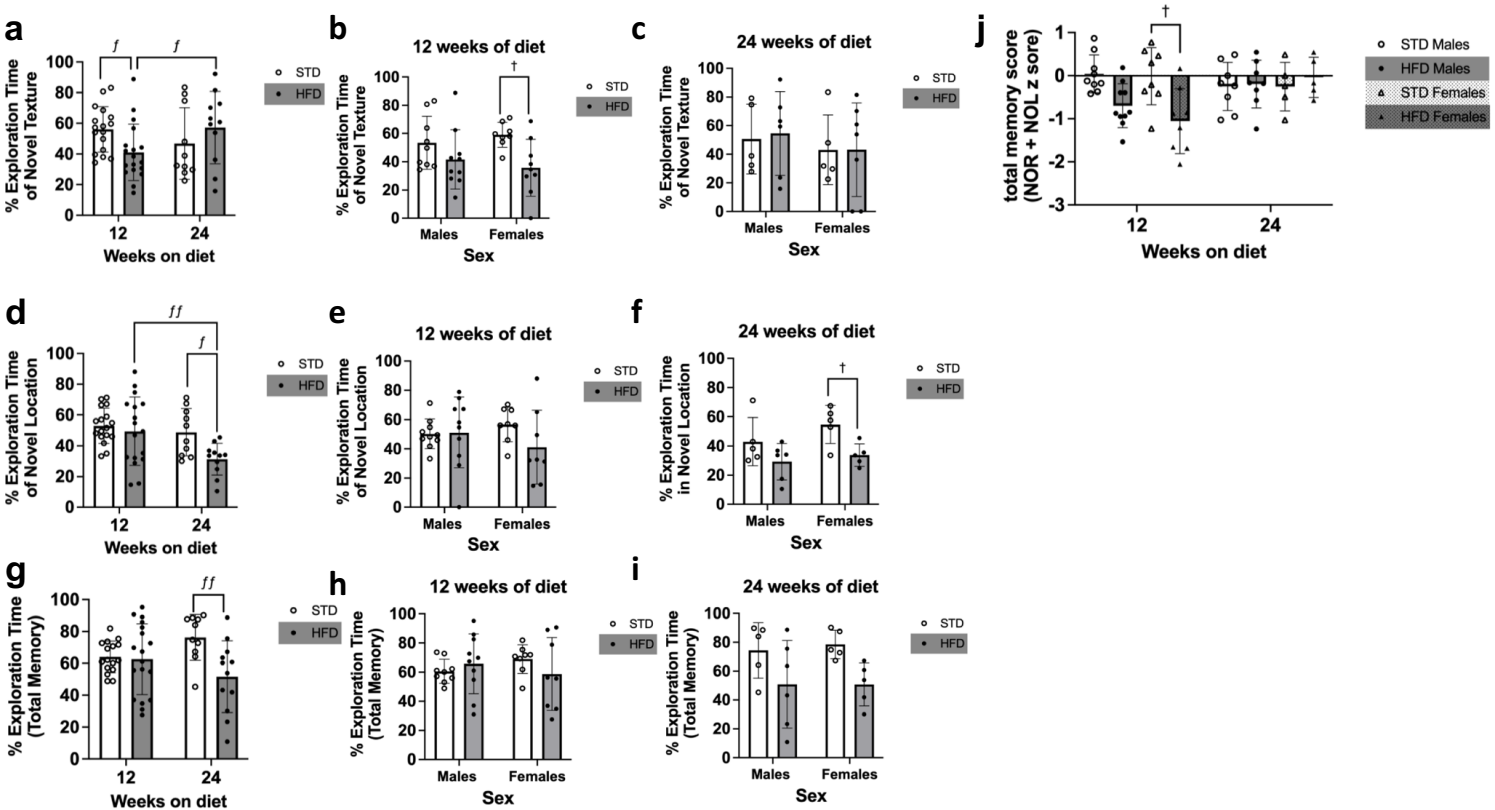
Task-Specific Behavioral Deficits in NOL and NTR are Driven by Diet. Assessment of diet duration and sex on NTR performance (**a**-**c**) and NOL measured by % exploration time of novel location (**d**-**f**) and total memory (**g**-**i**). Cumulative NOL and NTR z score after 12 and 24 weeks of diet (**j**). Data represented as mean ± SD. Data were analyzed using two-way ANOVA with time on diet or sex and diet as factors, followed by Uncorrected Fisher’s LSD or Šídák’s multiple comparisons test. *p* < 0.05 (*), *p* < 0.01 (**), *p* < 0.001 (***), *p* < 0.0001 (****). ƒ, †, and # indicate post hoc comparisons for combined-sex, female-only, and male-only groups, respectively; the number of symbols reflects the same *p*-value thresholds as asterisks. n $\:\ge\:$ 5

To determine whether diet influences cognitive performance in mid-life, male and female mice were assessed using the NOL and NTR tasks following 12 or 24 weeks of diet (Fig. 2).

In the NTR task, HFD animals spent less time exploring the novel texture after 12 weeks of diet, indicative of impaired recognition memory (Fig. 2a, b). A significant main effect of diet was observed when sex was stratified (F(1, 32) = 8.348; Fig. 2b), with post hoc analyses revealing reduced novel texture exploration exclusively in females after 12 weeks of diet. No significant differences were detected in either sex after 24 weeks of diet (Fig. 2c).

Memory performance was also assessed using the NOL task, measured as percentage of exploration time of the novel location (Fig. 2d) and percentage of exploration time total memory (Fig. 2g). Both NOL metrics showed a significant main effect of diet (novel location: F(1, 51) = 5.596, total memory: F(1, 52) = 6.767; Fig. 2d & g). When sex stratified, diet significantly affected NOL performance only after 24 weeks of exposure (F(1, 17) = 9.580 (novel location), 7.905 (total memory); Fig. 2d). Post hoc analyses of pooled (Fig. 2d & g) and sex-stratified (Fig. 2e-f and h-i) data revealed that this effect was driven by females, who spent less time in the novel location following prolonged HFD exposure (Fig. 2f).

To integrate cognitive performance across tasks, a composite z-score was generated from NTR and NOL outcomes. Diet significantly contributed to z-score variability (F(1, 53) = 6.30), and post hoc analyses revealed a significant reduction in z-scores in HFD females after 12 weeks of diet compared to their control, consistent with impaired cognitive performance (Fig. 2j).

#### Time-Dependent Variability in Y-Maze Performance Without Sex- or Diet-Specific Effects

**Fig. 3:**
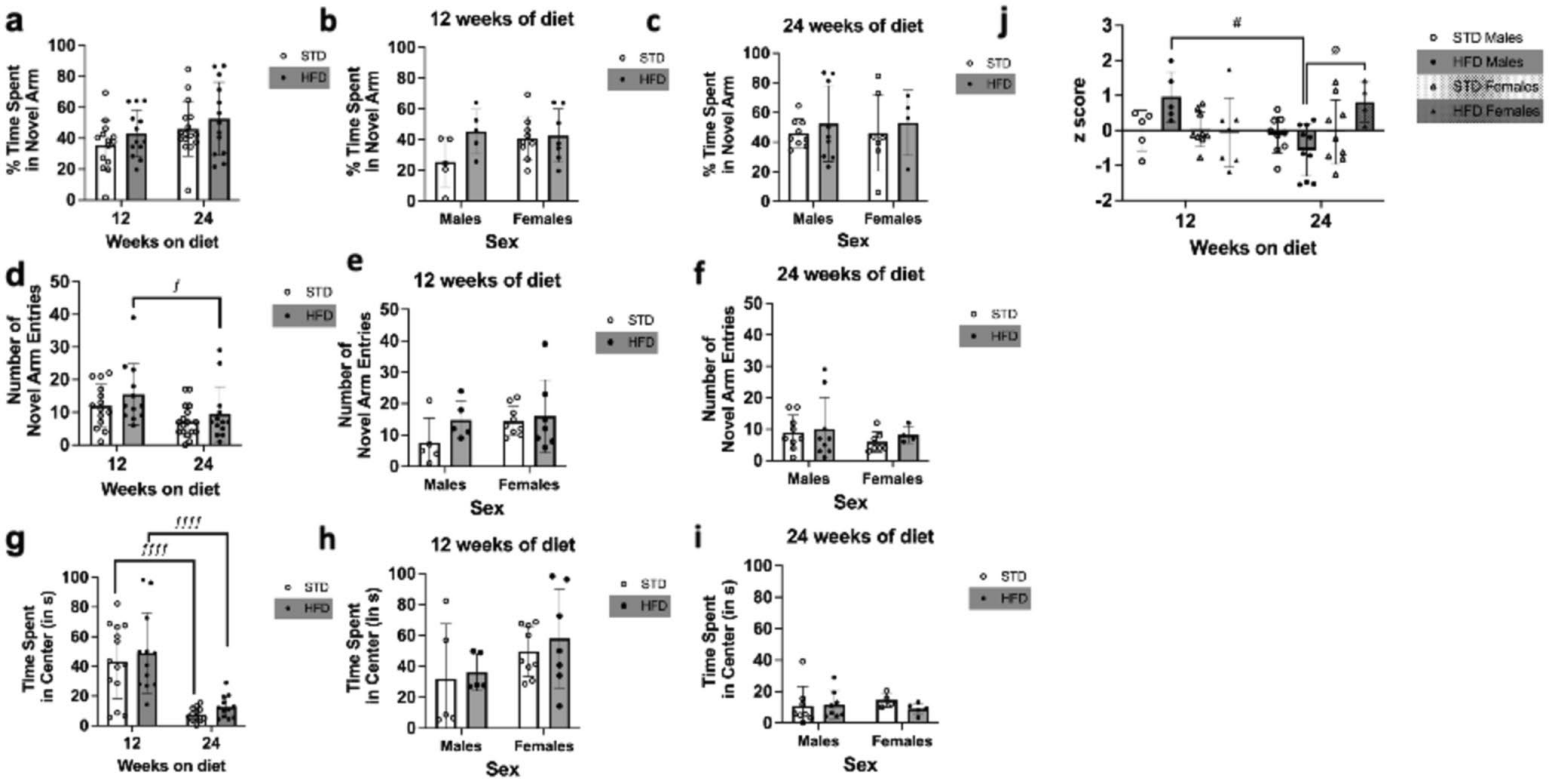
Time-Dependent Variability in Y-Maze Performance Without Sex- or Diet-Specific Effects. Y maze evaluation of % time spent in novel arm (**a**-**c**), number of novel arm entries (**d**-**f**), and time spent in center (**g**-**i**) with respect to time on diet and sex. Z-score analysis after 12 and 24 weeks of diet (**j**). Data represented as mean ± SD. Data were analyzed using two-way ANOVA with time on diet or sex and diet as factors, followed by Uncorrected Fisher’s LSD or Šídák’s multiple comparisons test. *p* < 0.05 (*), *p* < 0.01 (**), *p* < 0.001 (***), *p* < 0.0001 (****). ƒ, †, #, and ø indicate post hoc comparisons for combined-sex, female-only, male-only groups, and comparison between sexes, respectively; the number of symbols reflects the same *p*-value thresholds as asterisks. n $\:\ge\:$ 4

To evaluate whether dietary exposure affected cognitive domains beyond recognition memory, spatial working memory, and executive function were assessed using the Y-maze task (Fig. 3). Diet did not significantly influence the percentage of time spent in the novel arm (Fig. 3a) or the number of arm entries (Fig. 3d). However, time on diet did significantly affect both measures in sex-pooled data (F(1, 52) = 4.210 (% time), 7.549 (arm entries); Fig. 3a &d). No significant sex effects were detected at either 12 or 24 weeks, with sex stratification collapsing the effect of dietary duration (Fig. 3. e-f & h-i).

Indecisiveness was assessed by measuring the time spent in the maze center. Only the duration of the diet significantly affected time spent in the center (F(1, 46) = 43.54). Animals spent less time in the center over time (Fig. 3g). Composite z-score analysis demonstrated that time on diet and sex interacted to affect overall variability (F (1, 50) = 9.384). Post hoc analyses revealed that HFD males exhibited significantly poorer performance after 24 weeks of diet compared with HFD males at 12 weeks and with HFD females at 24 weeks of diet (Fig. 3j), suggesting that males exposed to prolonged dietary insults are uniquely susceptible to cognitive injury.

### Global Cortical Perfusion is Preserved Despite Region-Specific, Sex-Dependent Differences

**Fig. 4:**
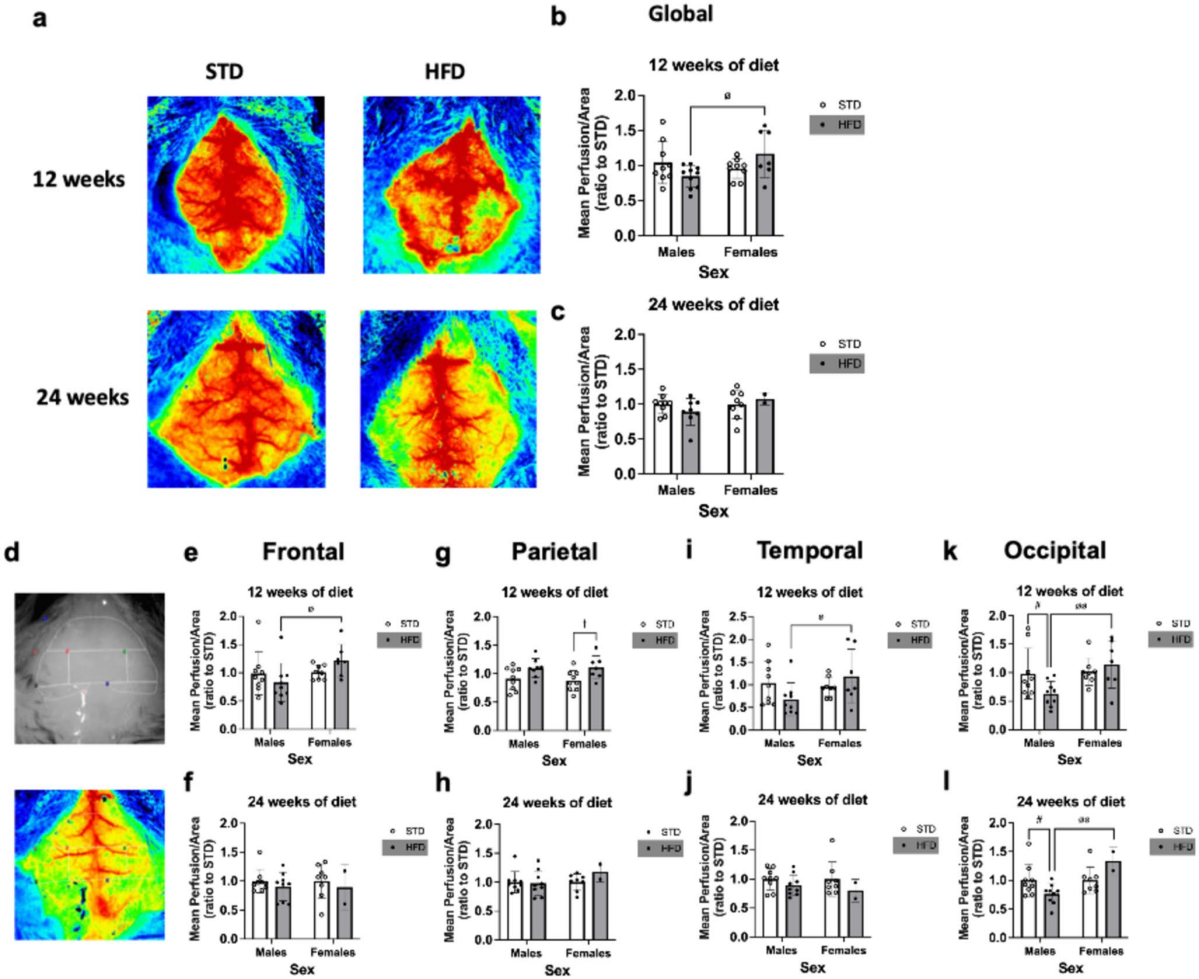
Global Cortical Perfusion is Preserved Despite Region-Specific, Sex-Dependent Differences. Representative cortical perfusion images after 12 and 24 weeks of diet (**a**). Global perfusion assessment after 12 and 24 weeks of diet (**b**, **c**). Schematic of brain regions and overlay with perfusion image (**d**). Brain region specific cortical perfusion after 12 and 24 weeks of diet separated by sex (**e**-**l**). Data represented as mean ± SD. Data were analyzed using two-way ANOVA with sex and diet as factors, followed by Uncorrected Fisher’s LSD. *p* < 0.05 (*), *p* < 0.01 (**), *p* < 0.001 (***), *p* < 0.0001 (****). ƒ, †, #, and ø indicate post hoc comparisons for combined-sex, female-only, male-only groups, and comparison between sexes, respectively; the number of symbols reflects the same *p*-value thresholds as asterisks. n $\:\ge\:$ 2

To examine diet-induced obesity associated cortical perfusion alterations, laser speckle contrast imaging was performed after 12 and 24 weeks of diet (Fig. 4a). Initial analyses detected no significant differences in global cortical perfusion following either 12 or 24 weeks of diet (Fig. 4b–c). However, a significant interaction was observed after 12 weeks of diet (F(1, 31) = 6.112), and post hoc analysis revealed reduced cortical perfusion in obese male mice compared to obese female counterparts (Fig. 4b).

After 24 weeks of diet, interpretation of sex-specific effects was limited by increased mortality in obese female mice during anesthesia, resulting in insufficient group size for reliable comparisons (Fig. 4c). Given that the study was not powered for sex-specific vascular outcomes, perfusion data were additionally analyzed with sexes combined; no significant diet-dependent differences were detected at either time point (Supplementary Fig. 1).

To assess region-specific perfusion changes that were masked in global analyses, cortical perfusion was further assessed using anatomically defined regional masks (Fig. 4d). After 12 weeks of diet, obese female mice exhibited significantly higher perfusion than obese males in the frontal (Fig. 4e), temporal (Fig. 4i), and occipital cortices (Fig. 4k). In the parietal cortex, diet significantly affected cortical perfusion at 12 weeks (F(1, 29) = 10.12), largely mediated by differences between obese and control females (Fig. 4g). Sex significantly contributed to perfusion variability in the occipital cortex at both 12 (F(1, 29) = 5.245; Fig. 4k) and 24 weeks of diet (F(1, 24) = 7.168; Fig. 4l), with post hoc analyses demonstrating reduced perfusion in obese male mice compared to both male controls and diet-matched females at each timepoint. No additional diet- or sex-dependent perfusion differences were observed in other cortical regions after 24 weeks of diet (Fig. 4f, h, j). We did not observe any differences in brain regions when combining sexes (Supplementary Fig. 1).

### Hippocampal Vascular Structure is Preserved Following Prolonged Dietary Exposure

**Fig. 5:**
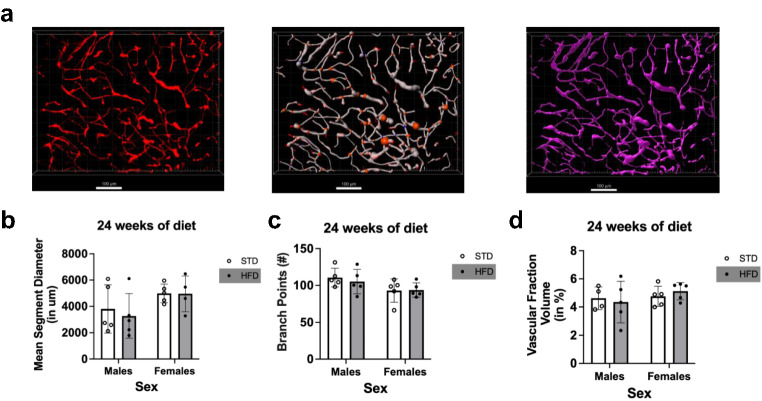
Hippocampal Vascular Structure is Preserved Following Prolonged Dietary Exposure. Representative image of lectin staining, branch point identification, and three-dimensional vascular network reconstruction (**a**). Quantification of mean vessel segment diameter (**b**), branch point number (**c**), and vascular fraction volume (**d**) in hippocampal vascular network at mid-life. Data represented as mean ± SD. Data were analyzed using two-way ANOVA with sex and diet as factors. *p* < 0.05 (*), *p* < 0.01 (**), *p* < 0.001 (***), *p* < 0.0001 (****). n $\:\ge\:$ 4

Given the hippocampus’ established vulnerability in dementia and the possibility of structural alterations independently of global perfusion changes, hippocampal vasculature was assessed via high-resolution 3D reconstruction (Fig. 5a). After 24 weeks of diet, no significant differences were detected in mean vessel segment diameter (Fig. 5b) or vascular fraction volume (Fig. 5d). Sex significantly contributed to variability in branch point number (F(1, 16) = 5.410; Fig. 5c); however, no diet-dependent effects were observed for this measure.

### Early-Life Obesity Differentially Reduces Cortical Perfusion Amongst Female Mice

**Fig. 6:**
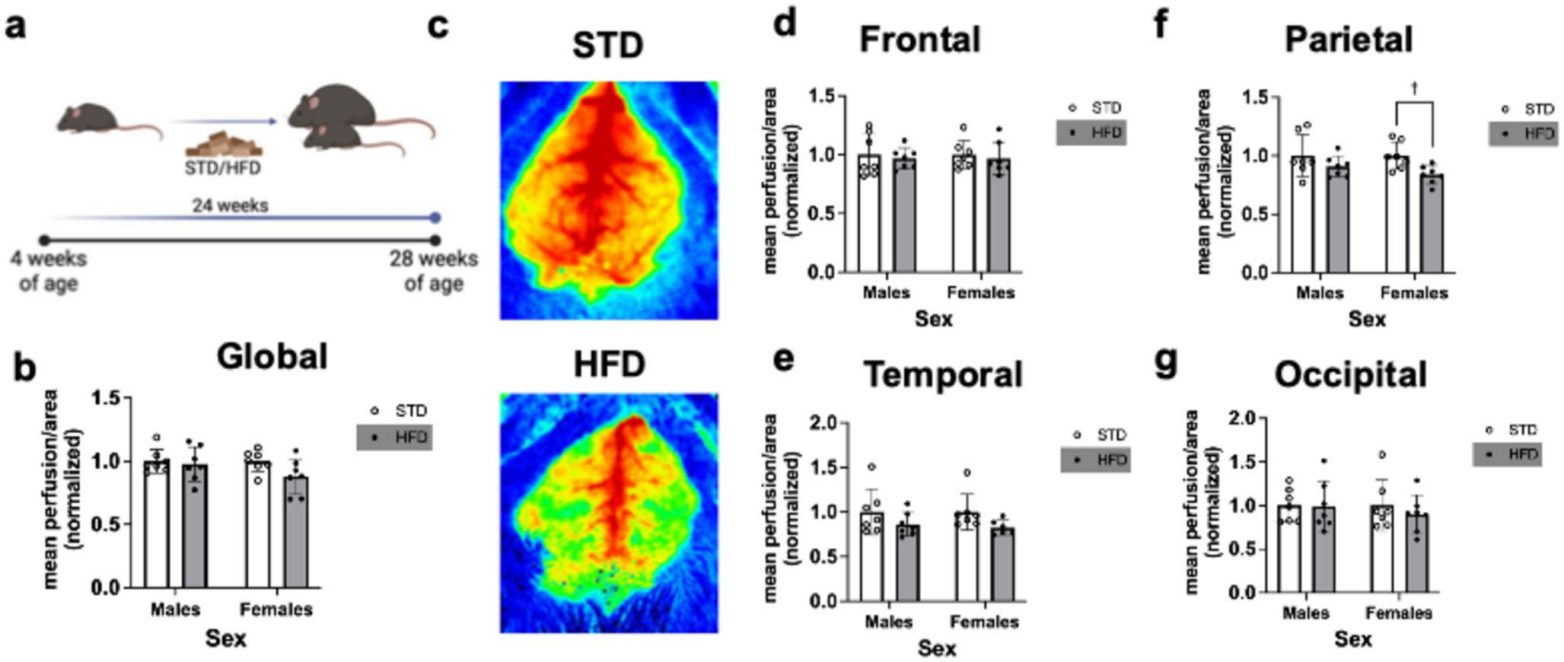
Early-Life Obesity Differentially Reduces Cortical Perfusion Amongst Female Mice. Schematic of early-life diet-induced obesity model (**a**). Global cortical perfusion following 24 weeks dietary exposure (**b**) with representative perfusion images (**c**). Region-specific cortical perfusion after 24 weeks of diet initiated in early life, shown by sex (**d**-**g**). Data represented as mean ± SD. Data were analyzed using two-way ANOVA with sex and diet as factors, followed by Uncorrected Fisher’s LSD. *p* < 0.05 (*), *p* < 0.01 (**), *p* < 0.001 (***), *p* < 0.0001 (****).† indicates post hoc comparisons for female-only comparison; the number of symbols reflects the same *p*-value thresholds as asterisks. n $\:\ge\:$ 7

Given the interactive effects seen on cortical perfusion following high-fat feeding in our mid-life obesity model (Fig. 4) and known cognitive deficits in mice subjected to an early-life diet-induced obesity model [19, 20], we next assessed whether obesity initiated earlier in life would result in more pronounced vascular effects. To evaluate the impact of prolonged dietary exposure beginning in adolescence, male and female mice were placed on HFD or STD for 24 weeks starting at 4 weeks of age (Fig. 6a), resulting in a comparable metabolic phenotype to our mid-life cohort (Supplementary Fig. 2). No significant differences nor effects were observed in global cortical perfusion amongst male and female mice (Fig. 6b).

Brain region stratification revealed a significant dietary effect on perfusion within the parietal (F(1, 24) = 7.517) and temporal cortices (F(1, 23) = 4.932). Specifically, obese female mice exhibited a significant decline in perfusion of the parietal cortex and a trending decline in perfusion of the temporal cortex compared to control counterparts (Fig. 6e&f). When sex-pooled, a significant decline in parietal perfusion is observed (Supplementary Fig. 3a).

### Early-Life Obesity Selectively Injures Hippocampal Vascular Architecture

**Fig. 7:**
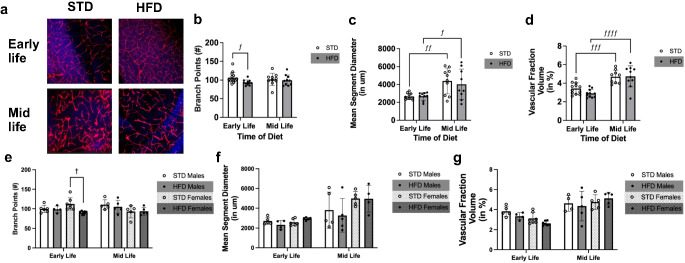
Early-life Obesity Selectively Injures Hippocampal Vascular Architecture. Representative images of hippocampal vascular following early- and mid-life diet-induced obesity (**a**). Quantitative comparison of branch point number (**b**), mean vessel segment diameter (**c**), and vascular fraction volume (**d**) between early- and mid-life. Effects of diet initiation timing and sex on branch point number (**e**), mean vessel segment diameter (**f**), vascular fraction volume(**g**). Data represented as mean ± SD. Data were analyzed using two-way ANOVA with time of diet and diet as factors, followed by Uncorrected Fisher’s LSD (b-d) or three-way ANOVA with time of diet initiation, sex, and diet as factors (**e**-**g**). *p* < 0.05 (*), *p* < 0.01 (**), *p* < 0.001 (***), *p* < 0.0001 (****), followed by Tukey’s HSD. ƒ and †, indicate post hoc comparisons for combined sex and female-only comparison; the number of symbols reflects the same *p*-value thresholds as asterisks. n $\:\ge\:$ 4

As significant and trending declines in perfusion were observed (Fig. 4, 6), we assessed hippocampal vascular architecture in mice after 24 weeks of HFD, starting at 4 weeks (early-life) or 16 weeks (mid-life) (Fig. 7). Diet-induced obesity initiated in early-life significantly reduced branch points amongst hippocampal vessels (Fig. 7b) and resulted in a trending decline in vascular fraction volume (Fig. 7d). The timing of diet initiation significantly affected both mean vessel segment diameter (F(1, 35) = 18.13; Fig. 7c) and vascular fraction volume (F(1, 38) = 44.00; Fig. 7d), with post hoc analyses revealing a significant decline in both metrics in our early-life model compared to mid-life counterparts. Mean vessel segment diameter (Fig. 7c) and vascular fraction volume (Fig. 7d) were also significantly higher amongst healthy controls following mid-life feeding.

Upon sex stratification, a significant effect was observed on mean vessel segment diameter (F(1, 31) = 6.114; Fig. 7f). Further, sex significantly interacted with time of diet initiation in the context of both branch points (F(1, 36) = 5.105; Fig. 7e) and vascular fraction volume (F(1, 34) = 6.455; Fig. 7g). Post hoc analyses unveiled a branch point reduction amongst early-life obese females compared to their control counterparts (Fig. 7e), suggesting that the observed significant and trending declines in parietal and temporal perfusion within this group, respectively (Fig. 6e, f), may be influenced by sparsity in vascular networks.

## Discussion

This study examines how diet-induced obesity across the lifespan affects metabolism, cognition, and brain vasculature, with attention to sex and age of onset. Our findings show that obesity-related effects are both task-specific and time-dependent, with early-life exposure producing more persistent vascular alterations than obesity in adulthood or mid-life. As child and adolescent brains exert higher metabolic demands [21], these findings reflect the heightened developmental vulnerability of the developing brain.

Consistent with prior work [20], HFD increased body weight and impaired glucose metabolism. Glucose dysregulation was sex-dependent, persisting in females but remaining transient in males, consistent with reports of age- and sex-specific metabolic vulnerability in diet-induced obesity [15, 22] and differing resilience to prolonged metabolic stress [23]. Notably, a clinical study linked higher fasting plasma glucose levels to hippocampal atrophy [24]. Together, these findings highlight the importance of considering sex as a biological variable when evaluating metabolic risk and its cognitive and vascular consequences.

Diet-induced obesity selectively impaired recognition memory (NOL, NTR) while sparing spatial working memory and executive function (Y-maze), suggesting preferential effects on hippocampal- and perirhinal-dependent processes. Recognition deficits were greater in females, particularly following prolonged dietary exposure, consistent with prior studies [22]. Although estradiol was not assessed in this study, its established positive association with cognition and dysregulated signaling in obesity underscores the possibility of hormone-driven sex differences [25, 26]. In contrast, Y-maze performance was unaffected by diet or sex, but varied with dietary duration, suggesting age- or experience-related effects rather than working memory impairment.

Cerebrovascular assessments revealed region-specific rather than global effects of obesity. While global cortical perfusion remained intact, regional analyses uncovered reduced occipital perfusion in males following adult-onset diet-induced obesity and reduced parietal and temporal perfusion in females exposed to obesity in early life. These regions, in particular the hippocampus, have high metabolic demands and may be especially vulnerable to inflammatory and metabolic stress on a sex-specific basis [27, 28]. Longitudinal studies support that early-adulthood obesity confers greater dementia risk than late-life obesity [29], supporting increased vulnerability to early metabolic insults. Future studies should determine whether similar deficits occur in white matter, a perfusion-sensitive tissue linked to cognitive decline [30].

Hippocampal vascular analyses revealed age-dependent vulnerability: mid-life obesity did not alter vascular structure, whereas early-life obesity reduced vascular complexity through decreased branching, vessel diameter, and vascular density. Sex also influenced vessel diameter and branching, consistent with reported region-specific differences [31]. These vascular changes may impair perfusion and neurovascular support within memory-related circuits, contributing to recognition memory deficits [20] and linking early life metabolic dysfunction to later cognitive decline. Diet-induced obesity has been further shown to differentially impact the expression of pro-inflammatory genes in an age-dependent manner [32], highlighting the need for future investigation into microglial activation and pro-inflammatory signaling in the brain. Future studies examining neurovascular coupling, blood-brain barrier integrity, synaptic function, and angiogenic signaling may further define these mechanisms and their sex-specific regulation [33, 34].

### Limitations

We acknowledge several limitations in this study. First, although sex-dependent effects and trends were observed across metabolic, cognitive, and vascular outcomes, the study was not powered specifically for sex-stratified analyses. Further, higher mortality in obese females reduced the sample size and limited conclusions about sex-specific vascular effects. Second, vascular measures were collected at discrete time points rather than longitudinally, thereby potentially missing dynamic changes in perfusion. Third, mechanistic links between metabolic dysfunction, vascular alterations, and cognition were not directly tested. Finally, early- and mid-life cohorts differed in age at study end (28 vs. 40 weeks; 24-week diet duration was chosen to align with prior work [19, 20]), introducing age as a confounding factor.

## Conclusions

This study provides evidence that diet-induced obesity exerts age- and sex-dependent effects on metabolism, cognition, and brain vasculature. Early-life obesity emerges as a particularly critical risk factor, producing persistent vascular changes and recognition memory impairments [19, 20], whereas mid-life obesity produces more selective and transient effects. Although some metabolic and cognitive measures improve following diet reversal [20, 35], it remains unclear whether vascular and structural changes can fully recover. The pronounced deficits following early dietary exposure [19, 20] suggest that metabolic stress during formative growth may limit later vascular plasticity, highlighting the importance of early intervention.

## Supplementary Information

Below is the link to the electronic supplementary material.

ESM 1 (DOCX 644 KB)

## Data Availability

All data and copyright are subject to the licensing and permission requirements of the VA and the United States government.

## Abbreviations

ANOVA: Analysis of Variance
GTT: Glucose Tolerance Test
HFD: High Fat Diet
HSD: Honestly Significant Difference
NTR: Novel Texture Recognition
NOL: Novel Object Location
SD: Standard Deviation
STD: Standard Diet

## Declarations

### Animal Studies

All institutional and national guidelines for the care and use of laboratory animals were followed and approved by the Ralph H. Johnson VA Medical Center Research and Development Committee (NURD-010–21 F, ACORP 712).

### Clinical Relevance

The perfusion patterns observed in this study parallel those in (1) Alzheimer’s disease, which predominantly affects women and is associated with reduced parietal and temporal perfusion [36, 37], and (2) dementia with Lewy bodies, which disproportionately affects men and correlates with occipital hypoperfusion [38, 39]. While risk prognostication for dementia sub-variants is beyond the scope of this study, these similarities highlight the utility of perfusion imaging and sex-specific analyses when investigating cognitive pathologies.
