# Supplementary material for: Timing and Duration of Prediabetic Metabolic Dysfunction Differentially Impact Cerebral Perfusion, Vascular Structure, and Behavior
Source: J Cardiovasc Transl Res. 2026 Aug 5;19(1):98. doi: 10.1007/s12265-026-10806-3 (PMC13442511; doi:10.1007/s12265-026-10806-3)
Supplement: Supplementary file 1 [file 12265_2026_10806_MOESM1_ESM.docx]

Supplemental Figures:

**Supplement Fig 1** Mid-Life Global and Cortical Perfusion (Sex-Combined)

Global cortical perfusion after 12 and 24 weeks of diet with sexes combined (a, b). Region-specific cortical perfusion after 12 and 24 weeks of diet with sex combined (c, d). Data represented as mean ± SD. n $\geq$ 10.

**Supplement Fig 2** Early-Life Body Weight and Fasting Glucose

Body weight (a) and fasting glucose (b) after 24 weeks of feeding with diet initiation at 4 weeks. Sex-stratified data in (c) and (d), respectively. Data represented as mean ± SD. Data (a, b) were analyzed using two-way ANOVA with diet and time as factors, followed by post-hoc analysis with Tukey HSD, unpaired t-test (c), or three-way ANOVA with diet, sex, and time as factors, followed by post-hoc analysis with Tukey HSD (d). p < 0.05 (*), p < 0.01 (****),** p **< 0.001 (*****), p < 0.0001 (****). ƒ, †, and # indicate post hoc comparisons for combined-sex, female-only, and male-only groups, respectively; the number of symbols reflects the same p-value thresholds as asterisks. n $\geq$ 4.

**Supplement Fig 3** Early-Life Global and Cortical Perfusion (Sex-Combined)

Global and region-specific cortical perfusion after 24 weeks of diet initiated at 6 weeks with sexes combined (a, b). Data represented as mean ± SD. Data were analyzed using unpaired t-test. p < 0.05 (*), p < 0.01 (****),** p **< 0.001 (*****), p < 0.0001 (****). n $\geq$ 13.
